# Active Ottumwa: Adapting Evidence-Based Recommendations to Promote Physical Activity in a Micropolitan New Destination Community

**DOI:** 10.3390/ijerph15050917

**Published:** 2018-05-04

**Authors:** Barbara Baquero, Christine M. Kava, Sato Ashida, Jason Daniel-Ulloa, Helena H. Laroche, Heidi Haines, Rebecca Bucklin, Adriana Maldonado, Mayra Coronado Garcia, Sandy Berto, Dan Sewell, Nicole Novak, Kathleen Janz, Claudia Gates, Edith A. Parker

**Affiliations:** 1 University of Iowa Prevention Research Center, Department of Community and Behavioral Health, University of Iowa College of Public Health, 145 N. Riverside Dr., Iowa City, IA 52240, USA; christine-morris@uiowa.edu (C.M.K); sato-ashida@uiowa.edu (S.A.); jason-daniel-ulloa@uiowa.edu (J.D.-U.); heidi-haines@uiowa.edu (H.H.); rebecca-bucklin@uiowa.edu (R.B.); adriana-maldonado@uiowa.edu (A.M.); sandy-berto@uiowa.edu (S.B.); nicole-novak@uiowa.edu (N.N.); edith-parker@uiowa.edu (E.A.P.); 2 Department of Internal Medicine, University of Iowa Carver College of Medicine, 451 Newton Rd., Iowa City, IA 52242, USA; helena-laroche@uiowa.edu; 3 Department of Biostatistics, University of Iowa College of Public Health, 145 N. Riverside Dr., Iowa City, IA 52240, USA; mayra-coronadogarcia@uiowa.edu (M.C.G.); daniel-sewell@uiowa.edu (D.S.); 4 Department of Health and Human Physiology, University of Iowa College of Liberal Arts and Sciences, 240 Schaeffer Hall, Iowa City, IA 52242, USA; kathleen-janz@uiowa.edu; 5 Community Advisory Board representative, Ottumwa Prevention Research Center office, 205 E. Main St., Ottumwa, IA 52556, USA; claudia.gates@usbank.com

**Keywords:** physical activity, intervention, community prevention, lay health advising

## Abstract

*Background*: Evidence-based interventions have been developed and tested to promote physical activity, but fewer studies have focused on identifying effective intervention strategies for mid-size rural communities, especially new immigrant destinations. We report here on the design and implementation of Active Ottumwa, a community-wide intervention using a lay health advisor approach to increase physical activity in a micropolitan new destination community in the rural state of Iowa. *Methods*: The Active Ottumwa study is part of a community-academic partnership in Ottumwa, IA. Evidence-based strategies recommended by the Community Guide for Preventive Services guided study implementation and included behavioral and social, campaign and informational, and environmental and policy approaches. Evaluation methods for this study are multi-faceted and include a cross-sectional community survey, longitudinal cohort assessment, observational data, key informant interviews, and project records. *Results*: We are currently in our second year of intervention implementation, with 45 lay health advisors (termed physical activity leaders here) trained to carry out behavioral and social intervention approaches, including walking groups, tai chi, and yoga. We have completed a communication and informational campaign utilizing five channels. Our longitudinal cohort has been recruited, with baseline and 12-month data collection completed. *Conclusions*: This study will assess the effectiveness and impact of a community-wide intervention to support physical activity.

## 1. Introduction

The Centres for Disease Control and Prevention recommend that adults obtain at least 150 min of moderate-intensity physical activity (PA) each week [1]. Despite advancements in health promotion efforts, many individuals and communities are not meeting these recommendations. According to data from the 2016 National Health Interview Survey, only 52% of adults are meeting PA guidelines [2]. An inactive lifestyle can lead to several negative health consequences, including increased risk for cardiovascular disease [3,4], type 2 diabetes [4], and all-cause mortality [3,5]. Furthermore, disparities in PA exist by race and ethnicity, income, and rural-urban residence. Nationally, African Americans [6] and Hispanics [7] are less physically active compared to whites. Compared to high-income residents living in suburban neighborhoods, low-income residents in rural communities are also less likely to meet PA recommendations [8].

Effective evidence-based interventions have been developed and tested to promote PA at the community level. These interventions have found improvements in PA [9,10,11,12], blood pressure [13,14], waist circumference [13,15], overall fitness [13], mental health [13,16], and perceived quality of life [17]. However, most of these interventions have been conducted in metropolitan (urban) areas. Fewer studies have focused on identifying effective interventions to promote PA in micropolitan, new destination communities. Micropolitan communities center around a population core of 10,000 to 49,999 people and are a relatively new classification of non-metropolitan areas developed by the U.S. Census Bureau [18]. New destination communities are outside of traditional immigrant-receiving regions in the southwest or borderland states that have received growing numbers of Hispanic immigrants since the 1990s [19].

While the focus has historically been on rural-urban differences in health, recent studies have found a smaller percentage of micropolitan residents meeting PA guidelines compared to residents living in more urban regions [20]. Immigrant population growth in new destination Midwestern areas, which are also often micropolitan communities [21], prompted by poor living and working conditions in traditional receiving communities [22], have created unique opportunities and challenges for addressing inequities in health. Research on evidence-based PA interventions that have been effectively adapted and implemented within these contexts is needed, given the health risks associated with physical inactivity and noted disparities.

In this paper, we report on the design and implementation of the Active Ottumwa (AO) study, a community-wide intervention to improve PA among residents of a micropolitan new destination community in the Midwest. A community-academic partnership between the University of Iowa Prevention Research Center (UI PRC) and a board of community leaders in Ottumwa, Iowa has guided the development and implementation of AO. This study is innovative because it advances our understanding on how to adapt and implement evidence-based community-wide interventions to promote PA in an understudied context and among a diverse population. While ongoing, our study has already addressed gaps in evidence related to (1) identifying effective interventions to promote PA in micropolitan, new destination communities, (2) evidence on how to effectively adapt and implement PA interventions within these communities and contexts, and (3) actively engaging with communities to determine how to adapt and implement intervention strategies to fit the needs of diverse populations.

## 2. Methods

### 2.1. Overview and Study Design

As briefly described above, the objective of the AO study is to determine the effectiveness of a community-based PA intervention. This intervention uses a lay health advisor (LHA) approach [23] to inform residents about PA, provide behavioral and social support, and advocate for social and environmental changes in a micropolitan, new destination community in the rural state of Iowa. Using a community-based participatory research (CBPR) approach, this intervention adapts and implements evidence-based strategies for PA as recommended by the Winnable Battles initiative [24] and the Community Guide for Preventive Services (CGPS) [25]. To comprehensively assess the effectiveness of the intervention, evaluation efforts take place at the individual-, community- and policy-level. To evaluate the implementation of the intervention, extensive measures of reach, dose, fidelity, acceptability and maintenance indicators are being collected [26]. Refer to Appendix A for a timeline of AO activities.

This study follows a Hybrid Type 1 design, allowing us to test the effectiveness of the intervention and assess the implementation of the intervention concurrently [27]. To examine the effectiveness of the intervention, we are conducting (1) a cross-sectional community survey, (2) a longitudinal cohort study with a sample of community residents to measure individual changes in PA, and (3) observational measures of PA in parks and open spaces. To assess the implementation of the intervention, we are (4) undertaking key informant interviews with local leaders and stakeholders and (5) collecting data on implementation, including measures of feasibility, dose delivered, cost, and sustainability. All study protocols were approved by the University of Iowa Institutional Review Board.

### 2.2. Study Foundation and Community Advisory Board

This study is part of a community-academic partnership between the UI PRC and organizations and residents of the city of Ottumwa, Iowa. The partnership uses a CBPR approach [28] in which representatives of community-based organizations, the public health department and agencies, and university representatives are involved as partners in all project components. The partnership began in April 2013 with the formation of a community advisory board (CAB), consisting of representatives from 12 community-based agencies and organizations, including the local school system, community college, United Way agency, community action agency, community health center, the YMCA, a local bank, and the City Parks Department, in addition to representatives from the UI PRC. The CAB decided to undertake a community health assessment to better identify health issues for potential intervention. After reviewing the data, we decided to focus on PA for the intervention.

### 2.3. Study Population

Ottumwa is a micropolitan community in Wapello County with a population of 24,487 [29]. Ottumwa is also a new destination community for Latinos and other immigrants, who have moved to Ottumwa from other countries or U.S. states. There has been a 1600% growth in Latino residents in Ottumwa between 1990 (200 Latino residents) to 2016 (3401 Latino residents), and Latinos now make up 14% of the town’s population [30]. Wapello County ranked 97 out of 99 Iowa counties in the latest Robert Wood Johnson county health rankings [31], with higher rates of premature death, obesity, and physical inactivity compared to the rest of Iowa. Ottumwa has higher rates of poverty (20.5%) compared to both Iowa (12.3%) and the United States (15.1%) [30].

### 2.4. Intervention

#### 2.4.1. Evidence-Based Physical Activity Interventions

**Figure 1:**
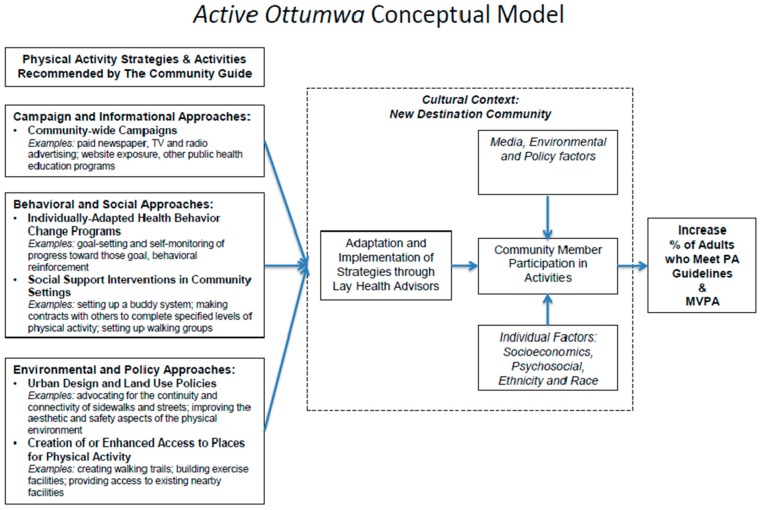
AO conceptual model.

Refer to Figure 1 to view the conceptual model for this study. The intervention was designed based on recommendations by the CGPS for PA [25]. We focused on the three main approaches recommended by the Guide: (1) behavioral and social strategies, which focus on individually adapted health behavior change programs and social support interventions in the community, (2) campaign and informational strategies, which refer to community-wide communication campaigns, and (3) environmental and policy strategies, focusing on community-scale design and land use policies to increase access to places for PA. Similar to previous studies [12,13,32,33], we decided to adopt a combination of approaches to increase the potential impact of intervention activities, and to determine their effectiveness in micropolitan communities.

#### 2.4.2. Adaptations

Adaptations of the intervention strategies from the three recommended approaches described above were needed to fit the community characteristics of Ottuwma, as most of the evidence-based strategies available in the CGPS have been tested and evaluated in urban contexts. To adapt the intervention strategies, we followed a systematic process. First, we reviewed the evidence to identify core intervention components. Then, we created a document that we called a “menu of activities” to share with members of the CAB. Adaptations were made considering the cultural, geographic, and social context of the community. For example, for cultural context we considered what it means to be a new destination community and the community identity. For geographic context, we considered seasonality, community size, and environmental resources (e.g., trails and parks). For the social context, we considered existing social networks and the use of social media.

Adaptations were based on four intervention components: (1) goals of the intervention strategy, (2) methods suggested, (3) execution of the intervention, and (4) channels of delivery [34]. We used an adaptation planning tool from the Cancer Prevention and Control Research Network [35] to help guide adaptations in terms of fit, acceptability, and the importance of each intervention strategy. The tool helped to identify modifications we should avoid, such as deleting important behavioral change constructs (e.g., reducing perceived barriers to PA), modifications to consider, such as sequence of activities, duration and who delivers the activities (e.g., LHAs), and modifications that are safe to be made, such as modifying images. A summary of potential adaptations were discussed and finalized in a series of meetings with CAB members to determine their feasiblilty for implementation. The CAB decided to deploy each recommended approach in sequence, starting with behavioral and social intervention strategies, followed by campaign and informational strategies, and finally environmental and policy strategies. We decided to use the LHA model during our design period to implement the behavioral and social approaches.

#### 2.4.3. LHA Model

LHAs have been previously defined as members of the community whom others go to for support and advice [36]. Acting as community change agents [37], LHAs leverage their social networks to influence the health-related attitudes, beliefs, and behaviors of others. Previous interventions using a LHA model have addressed several issues, including PA to improve fitness and health [13], HIV infection [38], and adolescent health [23]. AO is innovative in that few interventions have used this model across an entire community and among diverse populations. For this intervention, LHAs (termed physical activity leaders, or PALs, here) were recruited and trained to lead intervention activities.

We used multiple methods to identify and recruit PALs, including social network methods and other forms of recruitment such as the reputational method [36]. We primarily sought to recruit opinion leaders, or individuals in the community who have the capacity to influence the opinions, motivations, and behaviors of others [39]. To identify these people, we surveyed social group members at 14 different organizations in Ottumwa (e.g., churches, banks, etc.). In this survey, participants were asked to nominate up to five people within their social group who they went to for advice, for information about health, and whose opinions they trusted. Participants also checked off from a list of items characteristics they felt were important for a PAL to possess. This list was developed from previous research on LHAs [37,40,41,42,43,44,45,46,47,48,49,50,51] and included the following: “trusted”, “sensitive/warm”, “provides a lot of advice”, “good listener”, and “communicates well”.

Based on previous recommendations [52], we identified opinion leaders as nominees who received the top 10–15% of nominations within their social group. We also identified key players, or people who received less than 10% of all nominations but still received multiple nominations. These individuals may not be central figures in their social network but can play a key role in the diffusion of ideas [52]. We identified both groups based on nominations for each question (e.g., “Who do you go to for advice?”) and by examining nominations for multiple characteristics (e.g., those who received nominations for being “trusted” and as someone who “provides a lot of advice”). Once all surveys were completed, we sent letters to identified opinion leaders and key players inviting them to meet with us to discuss becoming a PAL.

#### 2.4.4. PAL Training and Support

The training for PALs was modeled after other effective LHA curricula [13,39,53,54]. The training program consisted of a two-day workshop (four hours total) designed to provide PALs with the skills needed to deliver intervention activities, and to enhance social support and teamwork among each other. During this training, PALs learned more about the UI PRC and AO, why we chose PA as a focal point for intervention, and core intervention strategies. PALs learned about the methods used to evaluate the AO project and the role that they played in this evaluation (e.g., submission of weekly activity logs). We also provided PALs with basic information on PA guidelines, levels of PA intensity, and injury prevention. At the end of training, AO staff assisted each PAL in developing goals for the program, including deciding on what activities they wanted to lead.

Although PALs are not paid a salary, they are awarded a stipend of up to $250 annually for training (e.g., Zumba certification) and for purchasing exercise equipment needed to carry out activities. PALs meet biweekly with the AO field coordinator to provide updates on their progress and activities. These meetings provide a designated time where PALs can share their feedback, ask questions and address concerns, facilitating open communication between PALs and AO staff. Other resources and support provided include educational materials on how to lead exercise groups, incentives for community members who participate in PAL activities, and smaller incentives such as t-shirts and water bottles.

#### 2.4.5. Campaign and Informational Approaches

Three strategies have been implemented as part of this approach: (1) a community-wide communication campaign, (2) a website and social media platform, and (3) face-to-face health education and promotion activities. The community-wide campaign was designed to increase program awareness and recognition, and to direct residents to our social media and website platforms for more information. The website and social media platforms offer daily updates on the program’s activities and connect PALs with their participants. PALs and intervention research staff conduct monthly outreach activities in the community to increase awareness of AO and educate residents on the benefits of physical activity.

The communication campaign used community-wide media channels to reach a large number of residents. The campaign lasted three months and utilized movie theaters, local radio, and TV stations to communicate messages related to the program. The website and social media platform continues to support the same messages. After the communication campaign, we created short videos about residents becoming active and the impact the program has had in the community. A series of print and digital materials were used to communicate monthly activities in the community and support engagement with the program.

#### 2.4.6. Environmental and Policy Approaches

We are currently planning for the implementation of strategies that promote policy and environmental changes in the community to support physical activity. The CAB wanted these efforts to be sustainable after the intervention grant ends. For example, we are in initial discussions with our community partners and some elected city officials about how the city of Ottumwa can manage and support Active Ottumwa activities. We are also developing activities to increase capacity and awareness for local government and stakeholders on policies and initiatives that promote active living and physical activity.

### 2.5. Evaluation

#### Primary and Secondary Outcomes

The primary outcome of this study is to increase the proportion of residents in Ottumwa who meet the PA guidelines for moderate-to-vigorous physical activity (MVPA), measured by self-report in the cross-sectional sample, and by both accelerometer and self-report in the longitudinal cohort sample. The secondary outcomes of this study include the following: (1) increase the use of parks and recreational facilities by residents, (2) identify policies to support PA in the community by local stakeholders and government, and (3) demonstrate the feasibility of adapting and implementing evidence-based interventions in a micropolitan and new destination context.

### 2.6. Individual-Level Evaluation Procedures

Two individual-level evaluation methods are being used for this study: (1) cross-sectional community-wide surveys conducted in 2013 and 2018 and (2) a longitudinal cohort, who were recruited and completed baseline data collection before the intervention started in 2015. Below we describe in detail the procedures for each evaluation method.

#### 2.6.1. Cross-Sectional Community Surveys

Two cross-sectional community-wide surveys were planned for this study. The first survey was conducted in 2013 and the follow-up survey will be conducted in 2018. The surveys are conducted via random digit dial (RDD) with adults 18 or older. The 89-question survey assesses a variety of health-related behaviors, including quality of life, social support, neighborhood context, discrimination, and basic demographic information. For a detailed overview of cross-sectional and cohort measures, refer to Appendix B.

The 2013 survey was conducted to inform the planning of the current study. Approximately 4000 people were contacted and 1101 completed the survey for a response rate of 25.3%. The results from this survey showed that 26.7% of respondents reported less than 150 min of PA a week, and 33.4% of respondents lived in poverty. Given this information, we decided to focus our intervention efforts on PA, which can be promoted community-wide, tailored to diverse populations, and can benefit community members suffering from chronic diseases.

#### 2.6.2. Longitudinal Cohort

**Figure 2:**
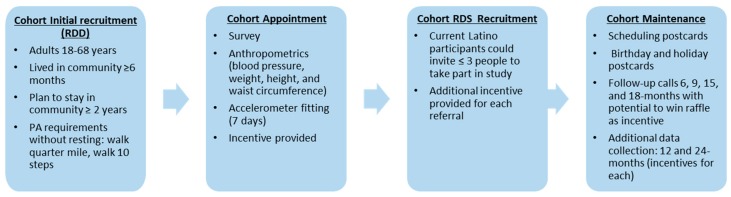
Longitudinal cohort recruitment, measurement, and maintenance procedures.

Longitudinal cohort recruitment began in October 2015. Community residents were contacted via RDD using both landlines and cell phones [55], and were screened to participate in the cohort (see Figure 2 below for details on study design and recruitment). We stratified the sample by gender and ethnicity to achieve a cohort that reflected the population distribution. Latino residents were oversampled by calling all cell phone numbers associated with a Latino surname. Interviews were conducted in English and Spanish as needed, with bilingual and bicultural staff interacting with respondents. We attempted to contact 4292 people and were able to assess that 222 were eligible. Of these 222, the UI PRC enrolled 142. To increase our Latino subsample size, we used respondent driven sampling (RDS). Latino cohort participants were eligible to serve as “seeds” and were informed that they could invite up to three people within their social network to participate in the survey, and were provided with an incentive for each referral. Out of the 142 individuals enrolled in the study, 13 were enrolled via RDS (May–June 2016) for a total Latino enrollment of 35. We collected data from the cohort at baseline, 12 months and 24 months.

Cohort maintenance and follow-up calls were conducted at six, 15 and 18 months. The calls served two purposes: to maintain our cohort and monitor awareness, and implementation of the intervention in the community. The calls consisted of checking contact information, questions about awareness of AO programming, and stages of change questions focused on PA. Baseline cohort surveys were conducted in English and Spanish. At the halfway point of the survey, blood pressure was measured. Following the completion of the survey, the research team took participants’ weight, waist circumference, and height. All measurements were taken twice to make sure that they were within 1 cm for height and waist, and 0.3 kg for weight [13,56]. If needed, a third measurement was taken. Participants received a copy of their measurements. Anthropometric measurement protocols were taken from the 2013 National Health and Nutrition Examination Survey Anthropometry Procedures Manual [57].

At the conclusion of the anthropometric measurements, participants were fitted with a GT3X+ triaxial accelerometer (Actigraph, Inc., Pensacola, FL, USA) [58] on their wrist to objectively measure our outcome PA measures. Waist belts were available for those whose job prohibited wrist wearing (e.g., meat packers working on the line). An extra wristband or belt was provided along with an activity monitor card and a postage-paid envelope to mail back the monitor. The activity monitor card was a written reminder of when to return the accelerometer but also a place for the participant to record if they needed to remove the monitor for a period of time. We asked participants to wear the monitor continuously (24/7) on their non-dominant wrist or waist for a week (seven days). We achieved 89% compliance at baseline, with most participants wearing the monitor an average of 7 days. This rate is adequate compared to similar studies [59,60].

### 2.7. Community Level Evaluation Procedures

#### 2.7.1. System of Observing Play and Recreation in Communities (SOPARC)

System of Observing Play and Recreation in Communities, or SOPARC, measures key characteristics of physically active residents and the context where this activity occurs [61]. Baseline observations were made in designated target areas that represented locations likely to provide opportunities for park users to be physically active [61]. Five parks and three trails were randomly selected for two weekdays and one weekend day in two to three hour segments [61]. Observations were made on adults and children, with data collected on an application designed for use with iPads (iSOPARC) [62]. Our research staff also made entries for time of day, area accessibility, area usability, presence of supervision, equipment, and presence and classification of organized activities. Follow-up observations have been conducted using similar methods at 12 months and are planned for 24 months. These measurements will help to determine park usage and the effects of the AO intervention.

#### 2.7.2. Rural Active Living Assessment (RALA)

RALA assesses the impact and change in the physical environment, town characteristics, community programs, and policies related to promoting PA [63]. This instrument consists of three components: (1) town-wide characteristics (18 questions) and an inventory about recreational amenities (15 questions), (2) program and policy (20 questions), and (3) street segment (28 questions). RALA data collection took place at baseline with organizational representatives (*n* = 7) including recreation facility managers, community-based organization leaders, and school district representatives. These representatives were interviewed to assess community resources and barriers that may be related to the adoption, implementation, and maintenance of the intervention. Sixteen street segment analyses were also conducted with a member of our CAB. Additional data collection will occur at 24 months to assess implementation, changes, and maintenance in the environment and policy to promote PA in the community.

### 2.8. Planned Analysis

#### 2.8.1. Cross-sectional Community Survey Data

The statistical analysis plan for assessing an intervention effect based on the 2018 cross-sectional data will use the PROC GENMOD procedure in SAS statistical software version 9.1.3. [64]. The magnitude of the intervention effects will be tested at a 0.05 level of significance. Subgroup analyses will also be performed for ethnicity and gender. The primary outcome will be binary, based on whether or not surveyed individuals meet the PA guidelines for MVPA. Change in the probability of meeting PA guidelines will be reported in magnitude and tested for statistical significance, accounting for any differences in the composition of the cross-section according to ethnicity, gender, or age.

#### 2.8.2. Longitudinal Cohort Data

The statistical analysis plan to assess an intervention effect based on the cohort data will follow a repeated measures analysis using the PROC MIXED and PROC GLIMMIX procedures in SAS statistical software version 9.1.3. [64]. The primary outcomes evaluated will be whether or not participants met the PA guidelines for MVPA according to the self-reported and accelerometer measures of MVPA. These outcomes are binary and recorded as repeated measures (baseline, 12 months, and 24 months). Changes in participants’ vital signs (e.g., systolic and diastolic blood pressures) or changes in weight and BMI will be reported and tested as a secondary assessment of an intervention effect. These changes will be reported in magnitude and tested for statistical significance against their baseline evaluations. Adjustments will be used to identify how the intervention effect is associated with other variables such as ethnicity, gender, demographics, health behaviors, reach index, psychosocial variables, and neighborhood characteristics. In the repeated measures analyses, we will use these variables as covariates and test for their significance and how they influence the intervention effect.

#### 2.8.3. SOPARC Analysis

The statistical analysis plan for the SOPARC data will also follow a repeated measures analysis using the PROC GLIMMIX procedure for count data. Primary intervention effects evaluated will be the number of physically active residents observed by gender, activity modes and levels, and estimated age, recorded as a repeated measure (baseline, 12 months, and 24 months).

#### 2.8.4. Qualitative Data Analysis of RALA Interviews

The key informant interviews from RALA data collection were audio-recorded and transcribed verbatim. The interviews will be analyzed using a focused coding process [65]. Focused code categories that exemplify specific themes that emerge from the coding process will be developed. Interviews will be coded in QSR NVivo [66], a qualitative data management program, to identify interconnectedness of salient themes.

#### 2.8.5. Intervention Implementation

Descriptive statistics such as means, frequencies, and percentages will be applied to determine reach rates, recruitment rate, percent of intervention implementation, and adoption. *t*-tests and mean comparisons will be used to examine differences between settings, venues, and subgroups of the population. Linear and logistic regression will be performed to identify correlates of implementation, reach, and adoption of the intervention.

### 2.9. Power Calculations

The power to detect the effects of the intervention were calculated based on the longitudinonal cohort. Based on our sample size calculations using the primary outcome of MVPA, and taking into account PA data in the United States measured by accelerometer [67], the estimated average daily MVPA is 32.16 min/day among males aged 17–70, with an estimated standard deviation of 1.8 min/day. MVPA differs according to ethnicity and gender, however, with the gender difference greater than that reported for ethnicity [68]. Given this, we considered gender as a factor for statistical design purposes. A repeated measures design with one between factor (gender) and one within factor (time: baseline, 12 months, 24 months) will have sufficient statistical power to detect change with two groups (male, female) of 65 participants each for a total 130 participants. The design achieves 88% power to test for a gender effect if the Geisser-Greenhouse Corrected F test is used with a 5% significance level and the actual effect standard deviation is one (an effect size of 0.28). This effect size results from the hypothesis that the intervention promotes an increase in the average daily MVPA by 10% from baseline at 12 months and an additional 5% increase between 12 and 24 months.

## 3. Results To Date

We are currently in year two of the intervention. We have trained 45 PALs for AO, 30 of which have led at least one activity in the community and 17 who are currently active. The retention rate for the first intervention year was 57% (17/30 PALs). The majority the of 45 PALs trained are women (76%, *n* = 34). PALs commit between two to three hours a week to AO, leading at least one activity each week. We currently offer 12 different types of activities, including walking groups, strength-training classes, and yoga (refer to Table 1 below for a detailed description of PAL intervention activities). A major emphasis has been placed on walking groups, given that they are widely accessible, have a low risk for injury, do not require formalized training to implement, and can be maintained over time. All activities are free to participate in and are located in 10 indoor and outdoor settings across the community.

We completed a comprehensive communication and informational campaign that utilized five channels and has resulted in 200,000 points of contact (total opportunities to reach residents with our message over the campaign period). We have also recruited our longitudinal cohort (*n* = 142) and completed baseline and 12-month data collection. A majority of cohort members are female (66%, *n* = 94), with a mean age of 47 (SD = 14.12). The average number of years of residency in Ottumwa is 29 (SD = 19.38), with most cohort members owning an apartment or house (66%, *n* = 93). A majority of cohort members are married (51%, *n* = 73), white (80%, *n* = 113), employed (70%, *n* = 99), and have some college education (58%, *n* = 82). At baseline, cohort members were on average per day 337 min (SD = 209.9) sedentary, 77 min (SD = 112.9) moderately active, and 28 min (SD = 58.5) vigorously active. We used a Wilcoxon rank-sum test to compare the cohort baseline data to the 2013 cross-sectional survey GPAQ data for sedentary behavior and MVPA, with no statistically significant differences found (*p* = 0.251 and *p* = 0.089, respectively).

Analyses comparing the demographic composition of the longitudinal cohort to the total Ottumwa population found that the cohort was relatively comparable to the total population of Ottumwa in terms of race (80% vs. 88% white) [30]. The cohort contained a slightly higher proportion of adult women than the gender distribution of adults in the community as a whole (66% vs. 51%) [30]. Research on study recruitment has found that men are less likely to participate in research studies compared to women, particularly men of low socioeconomic status or who are in poor health [69]. We are currently collecting data for the 24-month follow-up. In Spring 2018, we will deploy the cross-sectional survey. We are also planning to collect SOPARC and RALA data at 24 months and continue to collect implementation data from the intervention.

## 4. Discussion

This paper describes the initial implementation of a community-wide evidence-based intervention to promote PA at the individual- and community-level. A core component of the intervention is the application of a LHA model to establish and support behavioral and social intervention strategies. In collaboration with a CAB, all intervention strategies and activities have been adapted to fit the context of Ottumwa, a new destination micropolitan community. We are using an innovative evaluation design to examine the effectiveness of intervention activities on individual and community PA levels, and on the implementation of intervention activities. We are advancing the knowledge on how to adapt and implement evidence-based community-wide interventions for diverse micropolitan communities, which have been underrepresented in research and underserved in public health practice.

### 4.1. Lessons Learned

The contextual characteristics of Ottumwa present unique challenges and opportunities for intervention implementation. The intersection of rurality, micropolitanism, and new destination in the Midwest generates different dynamics that are critical to understand and consider when implementing this type of intervention. We describe the main lessons learned so far below.

#### 4.1.1. Capacity and Resources of the Community to Implement the Intervention

We learned quickly during intervention implementation that we needed to focus on building capacity, or investigating and leveraging in resources available in the community. As expected for this type of community trial, resources from both researchers and communities are needed. However, in the case of AO, the resources and capacity of the community to implement and participate in the intervention were not readily available. For example, community organizations did not have LHA programs where potential PALs could be recruited for AO. Our year of intervention planning consisted mainly of identifying resources in the community, and educating and increasing awareness of program aims for stakeholders and leaders of the community.

#### 4.1.2. Implementing Intervention Timeline

We rolled out each of the three intervention approaches in phases to allow adequate time to conduct an iterative adaptation of intervention activities. For example, identifying, recruiting, and training PALs took more time than expected. The PAL training was adapted to accommodate the schedules and needs of community leaders being trained. Intervention activities were adapted to ensure that PALs felt confident and comfortable leading them. After establishing a core group of PALs, we implemented the community-wide informational campaign, and are currently developing the policy and environmental strategies. This sequential approach to intervention implementation has allowed the CAB and researchers to focus on building and implementing high fidelity and dose activities, adjusting and adapting activities to the context of the community.

#### 4.1.3. Supporting and Maintaining Physical Activity Leaders

We recognize that our PAL retention rate in year 1 is not optimal, but also know that maintaining volunteer LHAs can be challenging. One of the few studies examining retention among LHAs is a recent study looking at predictors of activity level and retention among African American LHAs promoting breast and cervical cancer screening [70]. This study found a 68% retention rate and of those retained, 37% reported low activity levels. Our retention rate is lower than this study, but the results from the study suggest that retention is a challenge in these types of volunteer lay health advisor studies.

We have implemented several strategies to improve retention rates. First, we have increased our support for PALs in the form of monthly PAL-led meetings, where they can share successes and challenges among themselves. We have also set up an internal e-bulletin board, where the PALs can communicate with each other and the AO field coordinator about challenges and suggestions for activity implementation. Lastly, we have frequently sponsored PAL appreciation events (e.g., appreciation dinners) to honor their work and to ensure that the wider community is aware of their efforts. Our retention rate for the current intervention year (year 2) has improved tremendously, from to 57% to 88%.

#### 4.1.4. Community Advisory Board Essential on the Adaptation and Implementation of Strategies

The CAB has been critical for the adaptation and implementation of the intervention. The broad representation of organizations and groups in the CAB provide a rich collection of experiences and opinions to consider when making adaptations to the intervention strategies and activities. Our CAB is also essential for resolving implementation issues. For example, based on other evidence-based interventions using a LHA model, we explored the idea of compensating PALs to incentivize their participation. The CAB disagreed with this approach, indicating that it would not work in Ottumwa nor reflect the original vision of the program (i.e., a volunteer group of health leaders). We then brainstormed other alternatives to incentivize and retain LHAs, which have been successful. This community-engaged approach has allowed us to ground each adaptation and implementation decision on the social, economic, geographical, political, and historical characteristics of the community. While sometimes challenging, this approach has allowed us to demonstrate that it is possible to achieve a balance between community context, resources, and needs while maintaining the integrity of evidence-based interventions.

#### 4.1.5. Validity of Measures for Micropolitan Rural Communities

While many of the measures selected for this study were validated in smaller rural settings, they may not fully capture the characteristics of micropolitan communities. Ottumwa is situated between a rural town and small city, with a growing immigrant population that is changing the socio-demographic profile of the community. Measures of physical characteristics to support PA in the community based on small rural towns do not necessary capture the subtle differences in the physical environment of micropolitan communities. For example, interventions have suggested installing sidewalks to increase walking, however in micropolitan communities in rural states this may be harder to do. We are collecting qualitative and quantitative data to fully examine how to adapt these measures and capture the context of these micropolitan communities.

#### 4.1.6. Ethical Implications

Implementing this community-wide intervention has provided the CAB and research team opportunities to discuss and deal with some ethical challenges. Among the most important are (1) how to reach and provide the intervention to diverse subgroups of the population (e.g., Latinos, elders, refugees, middle age men), (2) hiring and training community members as research staff who understand the contextual dynamics of the community, while at the same time protecting individual human subjects rights, and (3) how to deal with conflict demands and expectations from community members and organizations. We have addressed these challenges proactively and lean on our CAB to determine action steps. Our long-term partnership has provided the support and mechanisms to deal with the unique challenges that we have faced. Having CAB representatives endorse and promote the study throughout Ottumwa contributes to building support and trust in the project. Seeing trusted and long-time members of the community be part of the research team also provides additional assurance to study participants.

### 4.2. Strengths and Limitations

This study has several notable strengths. It is a community-engaged and community-wide intervention that is using robust methods to adapt and implement an evidence-based physical activity intervention and evaluate its effectiveness. The study uses several sources of data, including self-report and objective measures of PA. A limitation is that our quasi-experimental design does not control for all sources of bias that may affect the impact of the intervention, and we may have missed important factors when adaptating the intervention to the setting and population. Non-response or selection bias may be present, given the low response rate on the 2013 cross-sectional survey and the fact that not all people eligible to enroll in the longitudinal cohort did so. Furthermore, the small sample size and high proportion of females relevant to the general population in the longitudinal cohort makes it harder to generalize the findings from this study. However, our sampling and recruitment methods (e.g., random digit dial) alleviate the potential for bias and help to ensure that the results from this study are relevant to other populations.

### 4.3. Next Steps

#### 4.3.1. Activate Active Ottumwa Ambassadors (AOAs)

To continue to develop awareness and support for Active Ottumwa, we have made the decision to recruit additional LHAs as Active Ottumwa Ambassadors (AOAs). The role of an AOA will be to promote Active Ottumwa activities, advocate for Active Ottumwa programming, motivate friends and family to participate in intervention activities, and encourage participants to continue their involvement in Active Ottumwa. Recruitment of AOAs is currently underway and follows the same methods as PAL recruitment. AOAs will participate in a one and a half hour training program, where they will learn more about Active Ottumwa and current intervention activities.

#### 4.3.2. Development of a Toolkit to Promote PA in Micropolitan Cities

After evaluation and implementation data is collected, we will prepare a toolkit for micropolitan cities interested in promoting PA and active living in their communities. The toolkit will consist of resources and steps that local governments and community organizations can take to promote PA in their community. We plan to include a cost component, detailing budgeting and resources needed to implement an intervention like this in other communities.

## 5. Conclusions

Active Ottumwa addresses a significant gap on evidence-based interventions for promoting physical activity, and advances our understanding on how to adapt and implement these evidence-based interventions in micropolitan communities. This manuscript describes our study design and intervention plan to demonstrate how we are examining both the effectiveness and implementation of the intervention using community-based participatory research and population-based approaches. We are advancing knowledge of effective implementation of evidence-based interventions by demonstrating how to adapt and implement these interventions based on community assets and needs. We expect that the findings from this study can guide public health practitioners, researchers, and local government on how to effectively promote and support active living in their communities.

## Figures and Tables

**Table 1:** PAL intervention activities, July 2017–Feb 2018.

Activity	# of PALs Leading Activity ^1^	Total Hours of Activity Offered	Total # of New Participants
Strength training	4	42	269
Tai Chi	1	46	483
Walking groups	20	274	904
Water aerobics	2	50	489
Yoga	5	31	217
Zumba/Dance Fitness	3	41	175
Other 2	11	80	429

^1^ Numbers do not add up to 45 because some PALs did not lead any activities, while others led multiple activities. ^2^ Other activities include biking, square dancing, Body Groove, light stretching and Frisbee golf.

## Abbreviations

This paper uses the following abbreviations:

AO	Active Ottumwa
AOA	Active Ottumwa Ambassadors
CAB	Community advisory board
CBPR	Community-based participatory research
CGPS	Community Guide for Preventive Services
ISRC	Iowa Social Science Research Center
MVPA	Moderate to vigorous physical activity
PA	Physical activity
PAL	Physical activity leader
RALA	Rural Active Living Assessment
RDD	Random digit dial
RDS	Respondent driven sampling
REDCap	Research Electronic Data Capture
SOPARC	System of Observing Play and Recreation in Communities
UI PRC	University of Iowa Prevention Research Center

## Appendix A

	**Timeline**					
**Activities**	**2015**		**2016**	**2017**	**2018**	**2019**
Identify, recruit and train Physical Activity Leaders (PALs)	x					
Determine and adapt intervention strategies to Ottumwa	x					
Baseline, 12- and 24-month longitudinal cohort of measures		x	**x**	**x**	**x**	
Observations of trail and parks usage (SOPARC)		x				
Assessment of physical activity city environment (RALA)		x				
Intervention implementation			**x**	**x**		
Cohort maintanance (repeated at 6, 12 and 18 months)			**x**	**x**		
2018 Cross-sectional community survey					**x**	
Analysis and reporting (data analysis, reporting of results, Development of toolkit for use in other Iowa communities)						**x**

## Appendix B

		**Community-Wide Cross-Sectional Survey**	**Cohort Assessment**			**Community-Wide Cross-Sectional Survey**
**Construct**			**Baseline**	**12-Month Follow-Up**	**24-Month Follow-Up**	
**Health Related Quality of Life**						
	Perceived overall health	✓	✓	✓	✓	✓
	Physical and mental health	✓				✓
	Sleep	✓	✓	✓	✓	✓
	Depression	✓	✓	✓	✓	✓
**Health Care**						
	Insurance	✓	✓	✓	✓	✓
	Access to regular care	✓	✓	✓	✓	
	Past health conditions		✓	✓	✓	
**Alcohol and Drugs**						
	Alcohol consumption	✓	✓	✓	✓	✓
	Cigarettes and tobacco	✓	✓	✓	✓	✓
	Vaping					✓
**Nutrition**						
	Drink consumption	✓	✓			
	Fast food consumption	✓	✓			
	Fruit and vegetables	✓				
	Water		✓			
	Self-monitoring diet		✓			
	Food security	✓				
**Physical Activity**						
	Vigorous activity at work	✓	✓	✓	✓	✓
	Moderate activity at work	✓	✓	✓	✓	✓
	Vigorous activity for recreation	✓	✓	✓	✓	✓
	Moderate activity for recreation	✓	✓	✓	✓	✓
	Activity during travel	✓	✓	✓	✓	✓
	Sedentary behavior	✓	✓	✓	✓	✓
	Park usage		✓	✓	✓	✓
	Park identification		✓	✓	✓	
**Outcome Expectations**						
	Physical activity outcome expectations		✓		✓	
**Support for Physical Activity**						
	Physical activity social support		✓		✓	
	Environmental support		✓			
**Self-efficacy**						
	Physical activity self-efficacy		✓		✓	
**Motivators**						
	Motivators for physical activity		✓		✓	
**Barriers**						
	Barriers to physical activity		✓		✓	
**Social Norms**						
	Subjective norms		✓		✓	
	Descriptive norms		✓		✓	
**Costs of Physical Activity**						
	Economic costs		✓	✓	✓	
**Perceptions**						
Parenting			✓			
**Unintended Effects**						
	Exercise injury		✓	✓	✓	
**Anthropometric Measures**						
	Blood pressure		✓	✓	✓	✓
	Weight		✓	✓	✓	✓
	Height		✓	✓	✓	✓
	Waist Circumference		✓	✓	✓	✓
**Neighborhood Context**						
	Social cohesion	✓	✓			✓
	Aesthetics	✓	✓			
	Safety	✓	✓			
	Walkability	✓	✓			
	Food environment	✓	✓			
	Violence	✓	✓			
	Community involvement	✓				
	Neighborhood health priorities	✓				
	Spatial stigma					✓
**Community Health**						
	Health of community	✓				
**Social Support**						
	Social Support	✓				✓
**Psychosocial Stressors**						
	Vigilance		✓		✓	
	Discrimination	✓				✓
	Reasons for discrimination	✓				✓
**Acculturation**						
	Latino/Hispanic background	✓	✓			✓
**Generational and Immigration**						
	Country of birth	✓	✓			✓
	Time of residency in the U.S.	✓	✓			✓
	Parents’ nationality	✓	✓			✓
	Grandparents’ nationality		✓			
	Contact with native county	✓				
**Awareness of the Program**						
	Results of AO messages		✓	✓	✓	✓
